# Exploring Interpersonal Trauma and Post-Traumatic Stress Disorder Across Different Income Level Countries

**DOI:** 10.1192/j.eurpsy.2026.11998

**Published:** 2026-07-31

**Authors:** S. Ahn, B. Nam, S. S. Hong, D. H. Han

**Affiliations:** 1 Chung-Ang University, Seoul; 2 Dr. Nam’s Psychiatric Clinic, Chungju, Korea, Republic Of

## Abstract

**Introduction:**

The consequences of interpersonal trauma and post-traumatic stress disorder (PTSD) are significant individual and societal burdens worldwide.

**Objectives:**

Investigations aiming to clarify the social and economic factors that affect PTSD experiences and perceptions in low- and middle-income countries (LMICs) are scarce.

**Methods:**

We conducted a systematic review of studies on the types and prevalence of interpersonal traumatic events, prevalence of PTSD among individuals exposed to interpersonal trauma, and PTSD interventions in LMICs and compared to in high-income countries (HICs). We screened PubMed, ScienceDirect, and Web of Science for relevant literature according to the Preferred Reporting Items for Systematic Reviews and Meta-analyses (PRISMA) guidelines.

**Results:**

We reviewed 32 studies on interpersonal trauma types, PTSD prevalence, and intervention availability in LMICs. Our findings reveal interpersonal trauma exposure is related to elevated PTSD prevalence, particularly among women and youth in LMICs. Evidence-based interventions such as trauma-focused cognitive behavioral therapy and narrative exposure therapy have been implemented in LMICs; however, research on PTSD treatment remains limited. Compared to HICs, LMICs publish fewer studies and have more limited access to standardized PTSD interventions.

**Conclusions:**

This review highlights the substantial burden of interpersonal trauma in LMICs and low recognition of PTSD among populations who have experienced trauma. However, national and methodological differences may have confounded the findings. Future research should prioritize the development of culturally sensitive interventions and acquire localized knowledge to better detect and treat PTSD in people who have experienced interpersonal trauma.

**Disclosure of Interest:**

?. Ahn: None Declared, B. Nam: None Declared, ?. S. Hong: None Declared, D. H. Han Grant / Research support from: the Korean Culture, Sports and Tourism R&D Program through the Korea Creative Content Agency grant funded by the Ministry of Culture, Sports and Tourism in 2025

